# Improved sparse domain super-resolution reconstruction algorithm based on CMUT

**DOI:** 10.1371/journal.pone.0290989

**Published:** 2023-08-31

**Authors:** Zhiqing Wei, Yanping Bai, Rong Cheng, Hongping Hu, Peng Wang, Wendong Zhang, Guojun Zhang

**Affiliations:** 1 School of Mathematics, North University of China, Taiyuan, China; 2 Key Laboratory of Dynamic Testing Technology, School of Instrument and Electronics, North University of China, Taiyuan, China; Hainan Normal University, CHINA

## Abstract

A novel breast ultrasound tomography system based on a circular array of capacitive micromechanical ultrasound transducers (CMUT) has broad application prospects. However, the images produced by this system are not suitable as input for the training phase of the super-resolution (SR) reconstruction algorithm. To solve the problem, this paper proposes an improved medical image super-resolution (MeSR) method based on the sparse domain. First, we use the simultaneous algebraic reconstruction technique (SART) with high imaging accuracy to reconstruct the image into a training image in a sparse domain model. Secondly, we denoise and enhance the contrast of the SART images to obtain improved detail images before training the dictionary. Then, we use the original detail image as the guide image to further process the improved detail image. Therefore, a high-precision dictionary was obtained during the testing phase and applied to filtered back projection SR reconstruction. We compared the proposed algorithm with previously reported algorithms in the Shepp Logan model and the model based on the CMUT background. The results showed significant improvements in peak signal-to-noise ratio, entropy, and average gradient compared to previously reported algorithms. The experimental results demonstrated that the proposed MeSR method can use noisy reconstructed images as input for the training phase of the SR algorithm and produce excellent visual effects.

## Introduction

The use of computer tomography (CT) is a common imaging method in biomedical research that provides images of specific parts of the human body. A new breast ultrasound tomography system, which utilizes a circular array of capacitive micromechanical ultrasound transducers (CMUT), has the potential for various applications. Its circular structure and high sensitivity [1] make it ideal for breast imaging, and it is also safe for the human body.

Over the years, advancements in image reconstruction technology have been significant. Computer tomography algorithms can be categorized into analytical and iterative methods. Analytical methods, such as linear back projection (LBP) and filtered back projection (FBP), have faster processing times. Iterative methods, including algebraic reconstruction technology (ART), simultaneous algebraic reconstruction technology (SART), and simultaneous iterative reconstruction technology (SIRT), are better suited for noise reduction and limited data imaging. Ultrasound tomography algorithms based on CMUT are still in the early stages of research. In practical applications, mechanical errors can cause imperfect and unclean images, resulting in decreased image resolution and noisy images that may affect doctors’ assessments.

Super-resolution (SR) reconstruction is an important branch of contemporary computer vision research that uses software techniques to turn existing low-resolution (LR) images into high-resolution (HR) ones [2]. This can be done by reconstructing an HR image using multiple LR images or by processing a single LR image to create an associated HR image. This article focuses on the SR reconstruction of a single medical image. There are four primary categories of single image SR reconstruction algorithms: interpolation-based, reconstruction-based, deep learning-based, and sparse domain-based.

Bilinear interpolation is used in [3] for medical picture SR reconstruction based on interpolation to improve contrast, image resolution, and total acquisition time. In [4] the Bessel interpolation approach was employed for HR 3D picture reconstruction in 3D space. A multi-frame LR ultrasound-based image enhancement system is proposed in [5], which uses a bicubic interpolation of images. [6] proposes a new interpolation algorithm that combines two-dimensional filters with interpolation techniques to improve the resolution of interpolated images. A method [7] that blends interpolation with deep learning has been suggested and demonstrated promising results, indicating that this is a promising future direction. However, because the interpolation method only considers the gray value of the pixel closest to the sample point to be measured and does not consider the relationship between other pixel points and the overall image, the reconstruction is jagged and the detail part is unclear.

The image SR method based on reconstruction usually combines one or more priors, such as introducing gradient priors, total change, and edge priors in the algorithm to constrain the image and estimate the image [8]. The enhancement technology created on this basis has also yielded positive results [9–12]. However, the model optimization process is time-consuming and converges slowly, and the results of this reconstruction method are significantly influenced by prior knowledge.

Deep learning methods mainly use many image pairs to train networks, which can transmit HR results. The first application of deep learning in the field of SR reconstruction was proposed in [13], which provides an end-to-end SRCNN network. A wavelet frequency separation attention network (WFSAN) for medical image super-resolution is proposed in [14]. The DRLN network was proposed in [15], which provides the advantage of reducing computational costs. The Residual Dense Attention Network (RDAN) for super-resolution COVID-19 CT images is demonstrated in [16]. A new network is proposed in [17] by combining the Laplacian pyramid structure with dense networks to reconstruct clear and reliable medical HR images. Deep learning introduces artificial redundant information in deconvolution operations. At present, most reconstruction models use simple stacking of convolutional layers and first-order feature statistics for shallow feature extraction and require a large amount of data during the training phase. This method has weak model interpretation ability and high computational complexity [18].

The sparse domain-based SR reconstruction technique has demonstrated promising results in medical imaging applications. Compared with interpolation methods, this type of algorithm improves accuracy; Compared with deep learning methods, it does not require any additional external datasets and avoids inexplicability. During the training phase, the quality of the dictionary is crucial for a successful construction, which plays a significant role in the sparse representation. However, due to issues such as machine noise or poor accuracy of reconstruction algorithms, medical image reconstruction involves raw noise, and traditional sparse domain methods may learn about the artifacts and noise parts of the reconstructed image. As a result, researchers are focusing on how to obtain a good dictionary when there is noise in the reconstructed image. To address this, an improved sparse domain model-based medical image SR reconstruction algorithm MeSR was studied.

The contributions of this article are summarized as follows.

1. Due to the natural noise characteristics of medical images, the MeSR algorithm has been proposed for the SR reconstruction of individual medical images. Unlike traditional methods, it can achieve SR and denoising while processing noisy images.
2. The core idea of MeSR is to denoise and enhance the input image before obtaining detailed images during the training phase. However, in order to prevent the noise in the image from being enhanced, guided filtering is chosen for operation. The obtained new vector is used as a new HR block to learn the HR dictionary, which represents the texture of the HR block but also represents the noise-free texture of the LR block. This approach results in a more accurate reconstruction.
3. Experimental comparisons between MeSR and other algorithms were conducted on the Shepp Logan model and the k-wave based CMUT breast simulation model, which confirmed the effectiveness of the MeSR algorithm.

The article is organized into five sections, with Section 2 introducing basic knowledge of the CMUT system and algorithms, Section 3 introducing the MeSR algorithm, and Section 4 presenting experimental results and comparisons. Finally, the conclusion is presented in Section 5.

## Related works

### Introduction to CMUT ring arrays

**Fig 1:**
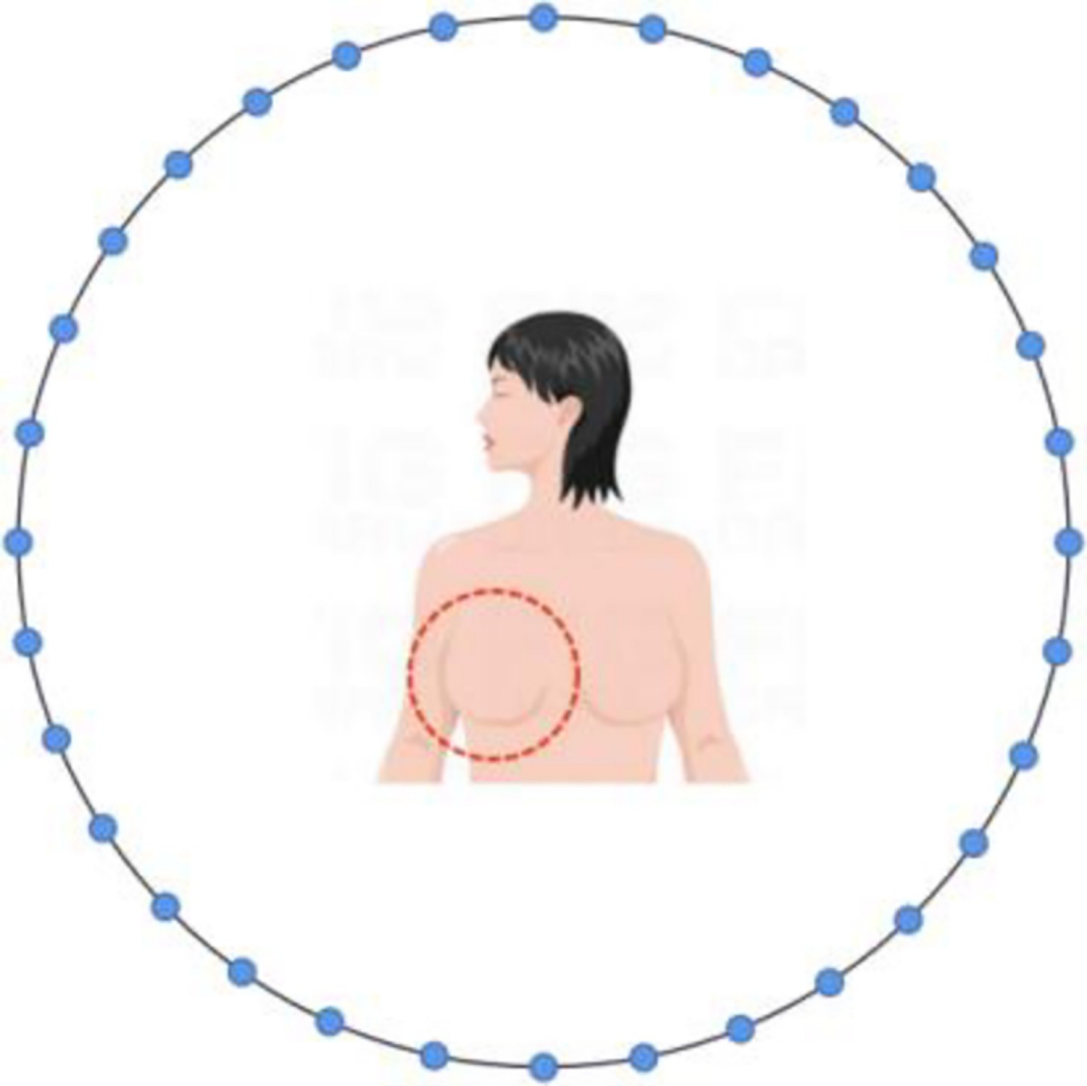
CMUT layout diagram display.

The breast ultrasound tomography system based on CMUT is distributed in a circular array at equal intervals around suspended breasts immersed in water. As shown in Fig 1, the ring is composed of equidistant units, each of which can independently transmit and receive ultrasonic signals.

In this simulation experiment, a single-cycle sine signal was used, with one transducer generating an emission pulse and all other transducers receiving ultrasonic signals. The interaction between the original signal and tissue and water, as well as geometric dilution, results in changes in the original signal upon arrival at the receiving element. Therefore, for each transmitted pulse, there is a set of received pulses with different shapes, amplitude, and arrival times. Measure the maximum ultrasonic pressure signal amplitude of the receiving transducer for constructing attenuation images.

*N* transducers have *N*(*N*−1)/2 independent receiving and transmitting pairs. Therefore, when *N* = 256, there are nearly 33000 receiving and transmitting transducer pairs in the system.

**Fig 2:**
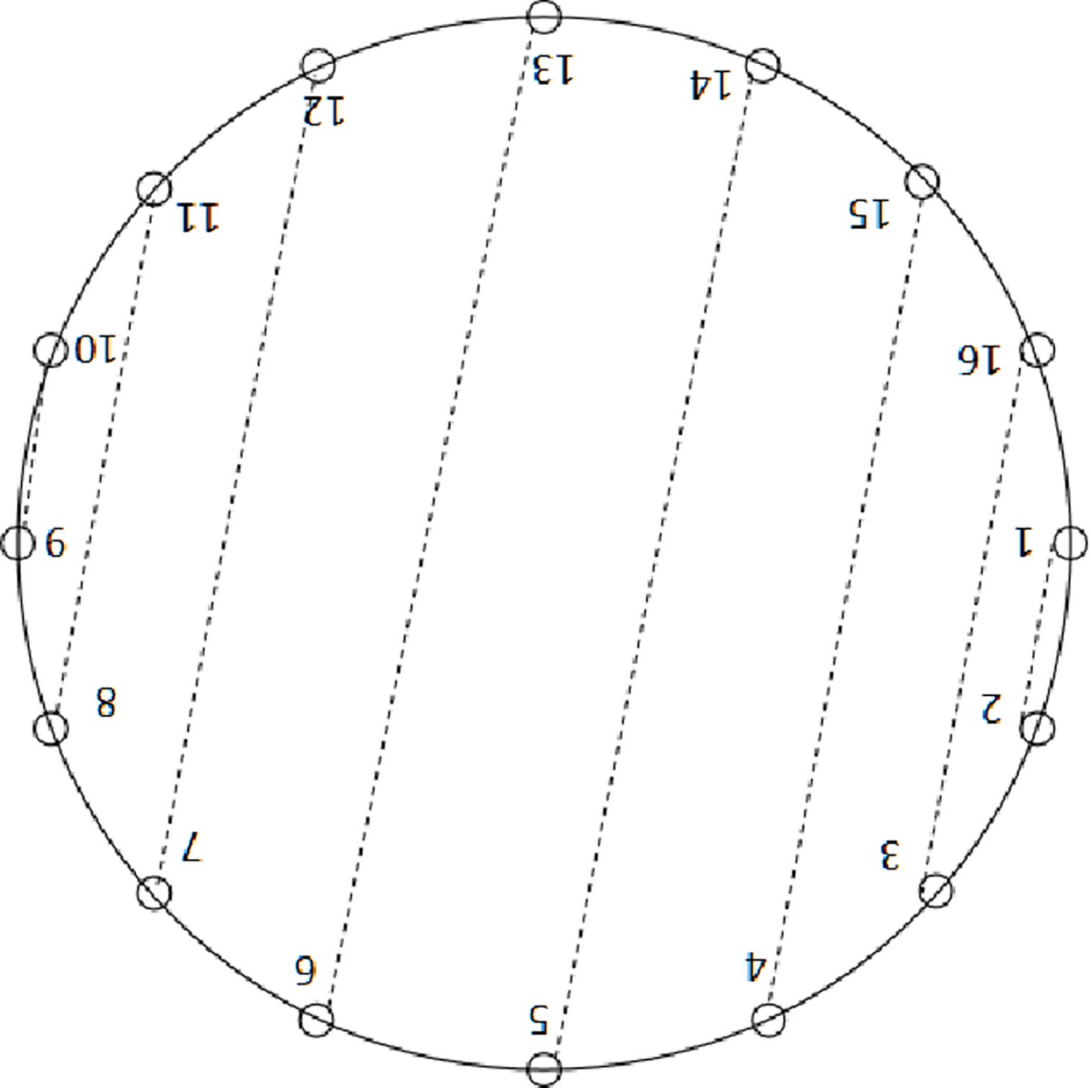
Using 16 transducers as a demonstration.

This article uses an inductive approach to rearrange the received data of 256 transducers, with transducer 1 transmitting and the other 256 transducers receiving as the first column of the matrix. Following this rule, a matrix of 256 columns can be obtained. According to the rules of this matrix, 256*256 data can be rearranged into 128*512 data. Afterward, the rearranged data can be supplemented, and cubic spline interpolation can be used to interpolate the data, resulting in equidistant parallel data. This article only studies CMUT ultrasound imaging at 128 angles, so data of 128*128 is obtained after equal interval sampling. The parallel data for 16 transducers is shown in Fig 2. At this point, we have obtained the parallel projection data required for ultrasound imaging, namely the sinogram.

### Radon transform and FBP reconstruction algorithm

The Radon transform [19, 20] was proposed by Austrian Mathematician Radon and is widely used in modern medical tomography. The Radon transform is useful because it can be used to find the line integral of an unknown two-dimensional distribution function in all directions. The Radon transform is written as

g(θ,r)=∬f(x,y)δ(xcosθ+ysinθ−r)dxdy, (1)

where *g*(*θ*,*r*) is the line integral of the image intensity, *f*(*x*,*y*) represents the unknown two-dimensional distribution function, *θ* represents the projection angle, *r* represents the distance from the origin, and the function *δ* serves to constrain *x* and *y* at this point to the line at the location of *r*.

FBP algorithm [21, 22], is a class of analytic methods in tomographic imaging. It is based on the central slice theorem, i.e., the one-dimensional Fourier transform obtained by projection of the null domain is a slice of the two-dimensional Fourier transform of the null domain. By taking all the projection values and then performing the inverse Fourier transform, the image of the spatial domain distribution is obtained, and a two-dimensional function can be obtained as

$$f(x,y)=\int_{0}^{\pi }\int_{-\infty }^{+\infty }S_{\theta }(\omega )|\omega |\times e^{2\pi j(x\cos\theta +y\sin\theta )}d\omega d\theta ,$$ (2)

where *S*_*θ*_(*ω*) is a two-dimensional Fourier transform of the projection under the angle *θ*, and its internal integration is the Fourier transform multiplied by |*ω*|, followed by an inverse Fourier transform. In the spatial domain, it represents the projection filtered by a function with a frequency domain response of |*ω*|. *q*_*θ*_(*r*) is used to represent this filtered projection.


$$q_{\theta }(r)=\int_{-\infty }^{+\infty }S_{\theta }(\omega )|\omega |e^{2\pi j(xcos\theta +ysin\theta )}d\omega ,$$ (3)

$$f(x,y)=\int_{0}^{\pi }{q_{\theta }(r)|}_{r=xcos\theta +ysin\theta }d\theta .$$ (4)

The above equation shows that the value of the reconstructed image *f*(*x*,*y*) at a certain location is a superposition of all the filtered projection samples through that point. Compared with the iterative reconstruction algorithm, the analytical method is less accurate but faster in terms of computational speed.

### SART reconstruction algorithm

The SART algorithm [23, 24] is a class of iterative reconstruction algorithms in tomographic imaging, which is based on the ART and SIRT algorithms and performs a single update of the projection data at only one projection sampling angle, i.e., the system equation corresponding to that projection angle. Before correcting pixel values, it is necessary to calculate the error of all rays passing through the pixel to correct the pixel and perform weighting and normalization. Then update the above results to the pixel and repeat the process until the convergence condition is met. The algorithm equation is

$$f_{j}^{\left(k+1\right)}=f_{j}^{\left(k\right)}+\frac{\sum_{p_{i}\in p_{L}}\left[\lambda \frac{p_{i}-\sum_{j=1}^{J}a_{ij}f_{j}^{\left(k\right)}}{\sum_{j=1}^{J}a_{ij}^{2}}\right]a_{ij}}{\sum_{p_{i}\in p_{L}}a_{ij}},$$ (5)

where *λ* is the relaxation factor, *k* is the number of iterations, *J* is the size of the image, *i* denotes the *i*^*th*^ ray, *j* denotes the *j*^*th*^ pixel point, $f_{j}^{\left(k\right)}$ denotes the gray value of the *j*^*th*^ pixel point at the *k*^*th*^ iteration, *p*_*i*_ denotes the true projection data of the *i*^*th*^ ray, *a*_*ij*_ denotes the length of the *i*^*th*^ ray intersecting the *j*^*th*^ pixel, and *p*_*L*_ is the set of the actual projection data at the same projection angle.

In terms of image reconstruction processing time, analytical methods have better performance than iterative algorithms. However, in terms of noise reduction and limited data, iterative methods outperform analytical methods. Therefore, in this article, we first use the SART reconstruction algorithm to obtain prior information on clearer images, and then use an improved sparse domain model to perform SR reconstruction of FBP images, ultimately obtaining HR images of FBP images.

### Improved sparse domain model

Single image SR reconstruction can be achieved using a sparse domain model-based method [25], which involves SR reconstruction of images using sparse representations.

Firstly, the features are extracted from the input image after local block processing. The LR image ***z***_*l*_ can be regarded as obtained from the HR image *y*_*h*_ by blurring and down sampling, i.e.

$$z_{l}=DVy_{h}+v,$$ (6)

where ***V*** denotes the blur operator, ***D*** denotes the down sampling operator, and ***v*** denotes the additive Gaussian white noise with mean 0 and standard deviation *σ*. The LR image ***y***_*l*_ is obtained by interpolating ***z***_*l*_ to recover the original scale size. Then we have

$$y_{l}=Ly_{h},$$ (7)

where ***L*** denotes the transform operator of ***y***_*l*_ obtained from ***y***_*h*_ through a series of transforms.

A local algorithm [18] is used for LR images ***y***_*l*_, i.e., the image is divided into local blocks $p_{k}^{l}$. For the HR image ***y***_*h*_ divided into local blocks $p_{k}^{h}$, assume that the sparse vector of $p_{k}^{h}$ on the dictionary ***A***_*h*_∈***R***^*n*×*m*^ is ***q***∈***R***^*m*^, where ‖***q***‖_0_<<*n*, i.e.


$$p_{k}^{h}=A_{h}q.$$ (8)

For the LR image block $p_{k}^{l}$, there are

$$p_{k}^{l}=Lp_{k}^{h}+{\tilde{v}}_{k},$$ (9)

where ***L*** is the local transformation operator, then multiplying both sides of (8) by ***L*** yields

$$Lp_{k}^{h}=LA_{h}q.$$ (10)

Therefore, there are

$$Lp_{k}^{h}=LA_{h}q=p_{k}^{l}-{\tilde{v}}_{k},$$ (11)

$${\left\|p_{k}^{l}-LA_{h}q\right\|}_{2}\leq \varepsilon ,$$ (12)

where *ε* is related to the power of the noise. The above procedure demonstrates that the recovery of $p_{k}^{h}$ can be done for a given LR image block using the training dictionary ***A***_*h*_.

**Fig 3:**
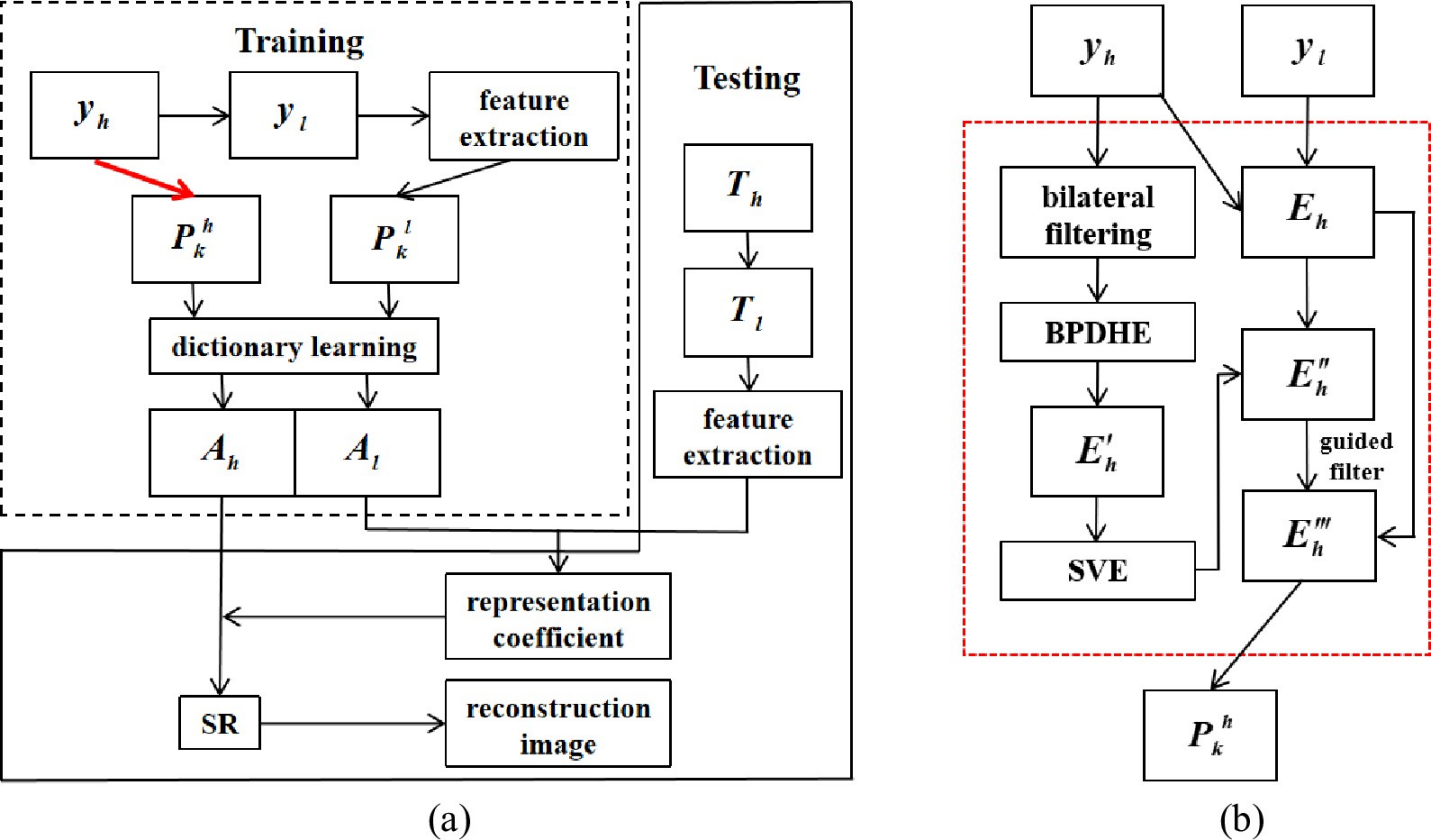
Schematic diagram based on the sparse domain model. (a) Original model;(b) Improved section.

Due to the unique nature of medical images, even HR images obtained through precise reconstruction algorithms still have a high possibility of artifacts and noise during the image reconstruction process. Therefore, this paper proposes to first use the SART algorithm, which has better imaging accuracy but slower imaging speed, to obtain the reconstructed image of the default Shepp-Logan model to imitate the HR image after the accurate reconstruction algorithm, i.e., the SART image pair set {***y***_*h*_,***y***_*l*_}. The red arrow in Fig 3 is improved by performing bilateral filtering on the above obtained ***y***_*h*_ to achieve edge-preserving and noise-reducing smoothing effects. However, since the bilateral filtering operation may produce an over-smoothing effect, resulting in ineffective detail information, Brightness Preserving Dynamic Histogram Equalization (BPDHE) is selected for the contrast enhancement operation [26]. BPDHE is obtained from dynamic histogram specification, which generates the specified histogram dynamically from the input image. Thus, $y_{h}^{\prime }$ can be obtained. The detailed image at this point is solved by

$$E_{h}^{\prime }=y_{h}^{\prime }-y_{l}.$$ (13)

Since singular value equalization (SVE) [27] can better preserve the quality and information content of the balanced image, the SVE operation is performed on the detailed map obtained above to obtain $E_{h}^{\prime \prime }$.


$$E_{h}^{\prime \prime }=SVE(E_{h}^{\prime }).$$ (14)

Due to the possibility of losing certain details during filtering operation, it is impossible to cover the complete original image information during dictionary training. Therefore, without filtering and contrast enhancement operations, ***y***_*h*_ is used to obtain ***E***_*h*_ with details and noise, which can be used as a guide image for $E_{h}^{\prime \prime }$ that may lose details [28]. Thus, we can obtain detailed images $E_{h}^{\prime \prime \prime }$ with more features and remove a large amount of artifact noise.


$$E_{h}^{\prime \prime \prime }=guidedfilter(E_{h},E_{h}^{\prime \prime }).$$ (15)

So, then the local algorithm is used to obtain the set of image block pairs with higher quality $\left\{p_{k}^{h},p_{k}^{l}\right\}$, and this set is used as the training set. Before learning the dictionary, a PCA dimensionality reduction [29] is performed to reduce the computational effort. The dictionary ***A***_*l*_ is trained for $p_{k}^{l}$ to obtain its sparse representation ***q***_*k*_. For the dictionary ***A***_*h*_, the solution is performed by

$$A_{h}=\arg\;\underset{A_{h}}{\min}\sum_{k}{\left\|p_{k}^{h}-A_{h}q_{k}\right\|}_{2}^{2}.$$ (16)

The above questions can be answered by

$$A_{h}=P_{h}Q^{+}=P_{h}Q^{T}{\left(QQ^{T}\right)}^{-1},$$ (17)

Where ***P***_*h*_ is the matrix consisting of the columns of ${\left\{p_{k}^{h}\right\}}_{k}$ and ***Q*** is the matrix consisting of the columns of {***q***_*k*_}_*k*_.

Pseudocode is shown in Algorithm 1.

*Algorithm 1*: The training stage in our algorithm MeSR

**Input:** Given the HR image ***y***_*h*_ and the corresponding LR image ***z***_*l*_.

**Output:** The dictionary ***A***_*h*_ and ***A***_*l*_.

1. Scale this image ***z***_*l*_ up and obtain ***y***_*l*_ by Eq (6) and Eq (7).

2. The pre-processing to obtain the detailed image $E_{h}^{\prime \prime }$: Perform bilateral filtering and BPDHE operations on ***y***_*h*_, then perform SVE operations on the resulting $E_{h}^{\prime }$, and then use the original ***E***_*h*_ to conduct a guided filter operation on the above obtained $E_{h}^{\prime \prime \prime }$ by Eq (15).

3. Extract the patches $p_{k}^{h}$ from $E_{h}^{\prime \prime \prime }$ of the location *k*.

4. Filter the image ***y***_*l*_ by using 4 high-pass filters.

5. Extract patches from the filtered images and concatenate the corresponding filtered patches into a vector. Each patch corresponds to ${\tilde{p}}_{k}^{l}$ from the location *k*; the PCA algorithm is used to reduce the dimensionality by multiplying the projection operator, resulting in the set $\left\{p_{k}^{l}\right\}$.

6. Form the training database ***P*** = [***P***_1_,***P***_2_,…,***P***_*J*_], and apply the K-SVD training procedure for the sample database ***P*** to obtain ***D***.

7. End

Then, the Shepp-Logan model with parameter changes is reconstructed using the FBP imaging algorithm. The imaging accuracy of this algorithm is not as good as the SART imaging algorithm, but the calculation speed is faster. The obtained FBP image is used as input for the testing phase. The FBP images are processed to extract local blocks to obtain the test dataset ${\left\{{\tilde{p}}_{k}^{l}\right\}}_{k}$, which is subjected to PCA dimensionality reduction, and then its sparse representation ${\tilde{q}}_{k}$ is found using the orthogonal matching tracking method (OMP) [30]. Using the LR dictionary ***A***_*l*_ obtained during the training phase and the LR image blocks at this time to obtain sparse representations ***Q***, and then using the HR dictionary ***A***_*h*_ to obtain the reconstructed HR image blocks of the FBP, after certain restoration steps, we can obtain the LR images of the FBP.

## Results

### Quantitative evaluation indicators

Select peak signal-to-noise ratio (PSNR) as the evaluation indicator for the algorithm’s image reconstruction in this article. The PSNR value is directly proportional to the reconstruction accuracy and is derived from the visual error between the original image and the reconstructed image. The formula is:

$$PSNR=10log_{10}\frac{MaxValue^{2}}{MSE},$$ (18)

where MSE is the mean square error, defined by the following equation:

$$MSE=\frac{\sum_{i=1}^{M}\sum_{j=1}^{N}{\left[f_{0}(i,j)‐f(i,j)\right]}^{2}}{M\times N},$$ (19)

where *M* and *N* denote the number of rows of the image, *f*_*0*_(*i*, *j*) and *f*(*i*, *j*) denote the pixel values of the original image and the reconstructed image at the location (*i*, *j*), respectively.

The average gradient (AG) reflects the rate of change in contrast to small details in an image, that is, the density change rate in the multi-dimensional direction of the image, and characterizes the relative clarity of the image. Usually, the larger the AG, the more hierarchical the image, and the clearer the image.

In SR reconstruction tasks, it has been found that high PSNR does not necessarily represent better reconstruction quality. Image entropy is a statistical feature, which reflects the richness of image information and the amount of average information from the perspective of information theory. Generally, the higher the image information entropy is, the richer the information is, and the better the quality is.

The following comparative experiments will be conducted on two models to compare the effectiveness of model reconstruction.

### Experimental results and analysis

**Fig 4:**
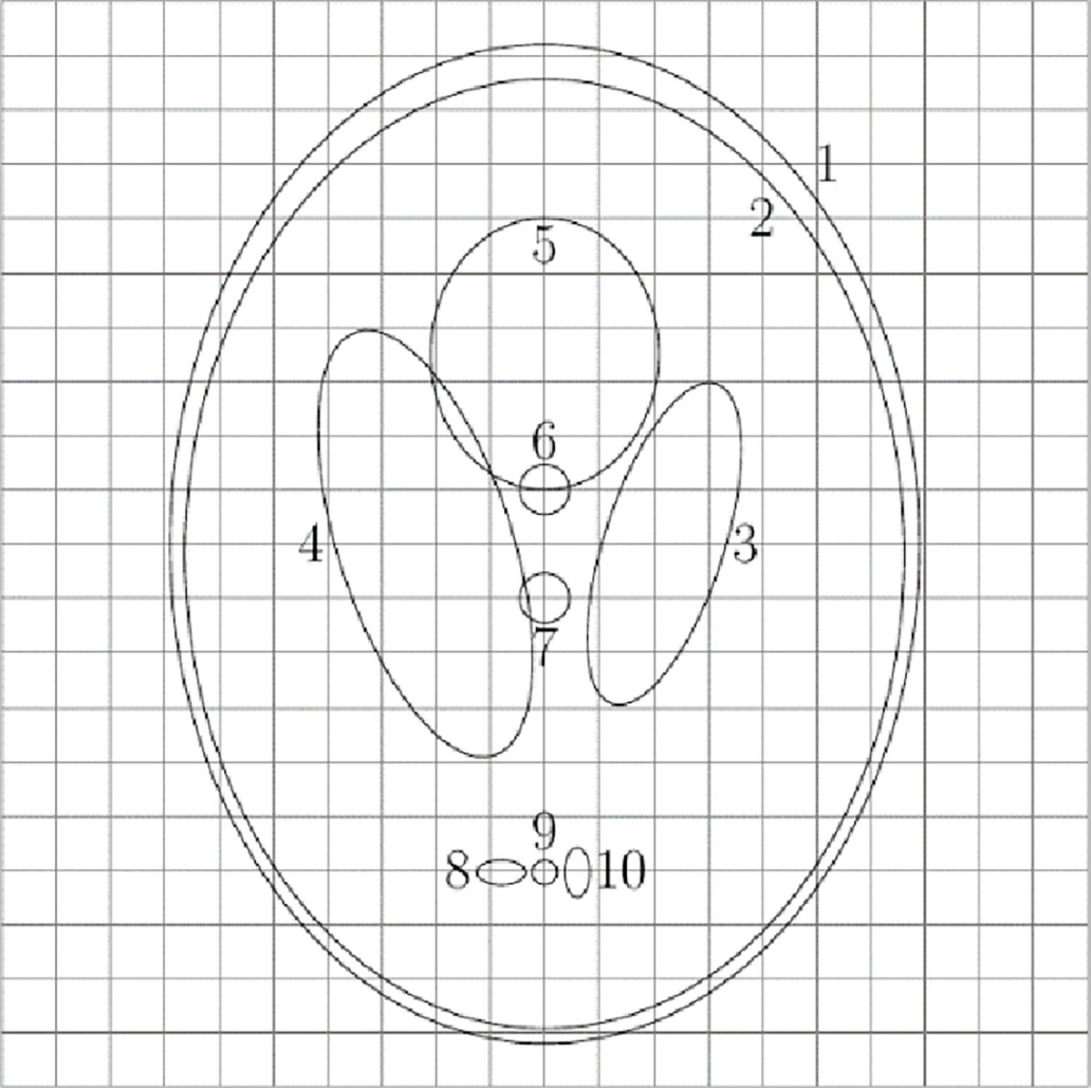
Shepp-Logan model.

**Shepp-Logan model reconstruction.** L.A. Shepp and B.F. Logan first developed the Shepp-Logan model [31] in 1974. It consists of 10 ellipses, each of which has size, orientation, density, and rotation angle that are all set by six default parameters, which are displayed in Table 1, and the model created using MATLAB is displayed in Fig 4.

**Table 1:** Shepp-Logan model parameters.

*N*_0_	*x*_0_	*y*_0_	*a*_*l*_	*b*_*s*_	*φ*	*ρ*_0_
1	0	0	0.6900	0.9200	0	1.0
2	0	-0.0184	0.6624	0.8740	0	-0.8
3	0.2200	0	0.1100	0.3100	-18	-0.2
4	-0.2200	0	0.1600	0.4100	18	-0.2
5	0	0.3500	0.2100	0.2500	0	0.1
6	0	0.1000	0.0460	0.0460	0	0.1
7	0	-0.1000	0.0460	0.0460	0	0.1
8	-0.0800	-0.6050	0.0460	0.0230	0	0.1
9	0	-0.6050	0.0230	0.0230	0	0.1
10	0.0600	-0.6050	0.0230	0.0460	0	0.1

**Fig 5:**
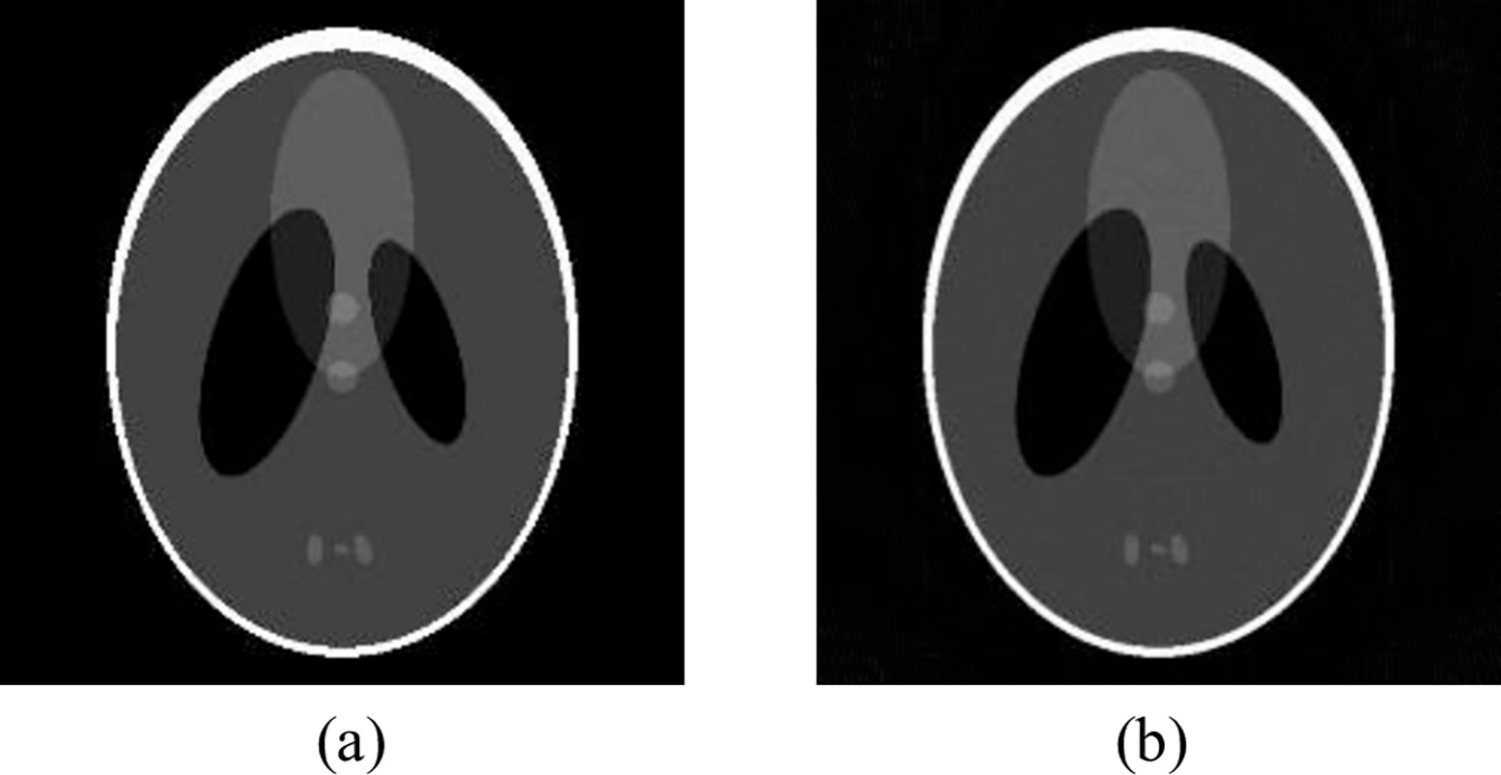
Shepp-Logan model and FBP reconstruction after changing parameters (a) Shepp-Logan model after changing parameters;(b) FBP image.

Compare our proposed MeSR algorithm with other algorithms in the literature, and then observe and analyze the reconstruction effect. To verify that this experimental model has some generalization, the input images of the training phase and the testing phase were somewhat differentiated, i.e., the relevant parameters of the skull model were changed before the FBP imaging, and the model parameters are shown in Table 2, the FBP reconstructed image is shown in Fig 5.

**Table 2:** Parameters setting after changing the Shepp-Logan model.

*N*_0_	*x*_0_	*y*_0_	*a*_*l*_	*b*_*s*_	*φ*	*ρ*_0_
1	0	0	0.6900	0.9200	0	1.0
2	0	-0.0184	0.6624	0.8740	0	-0.8
3	0.2200	0	0.1100	0.3100	18	-0.2
4	-0.2200	0	0.1600	0.4100	-18	-0.2
5	0	0.3500	0.2100	0.4500	0	0.1
6	0	0.1000	0.0460	0.0460	20	0.1
7	0	-0.1000	0.0460	0.0460	-10	0.1
8	-0.0800	-0.6050	0.0460	0.0230	90	0.1
9	0	-0.6050	0.0230	0.0130	-15	0.1
10	0.0600	-0.6050	0.0230	0.0460	15	0.1

**Fig 6:**
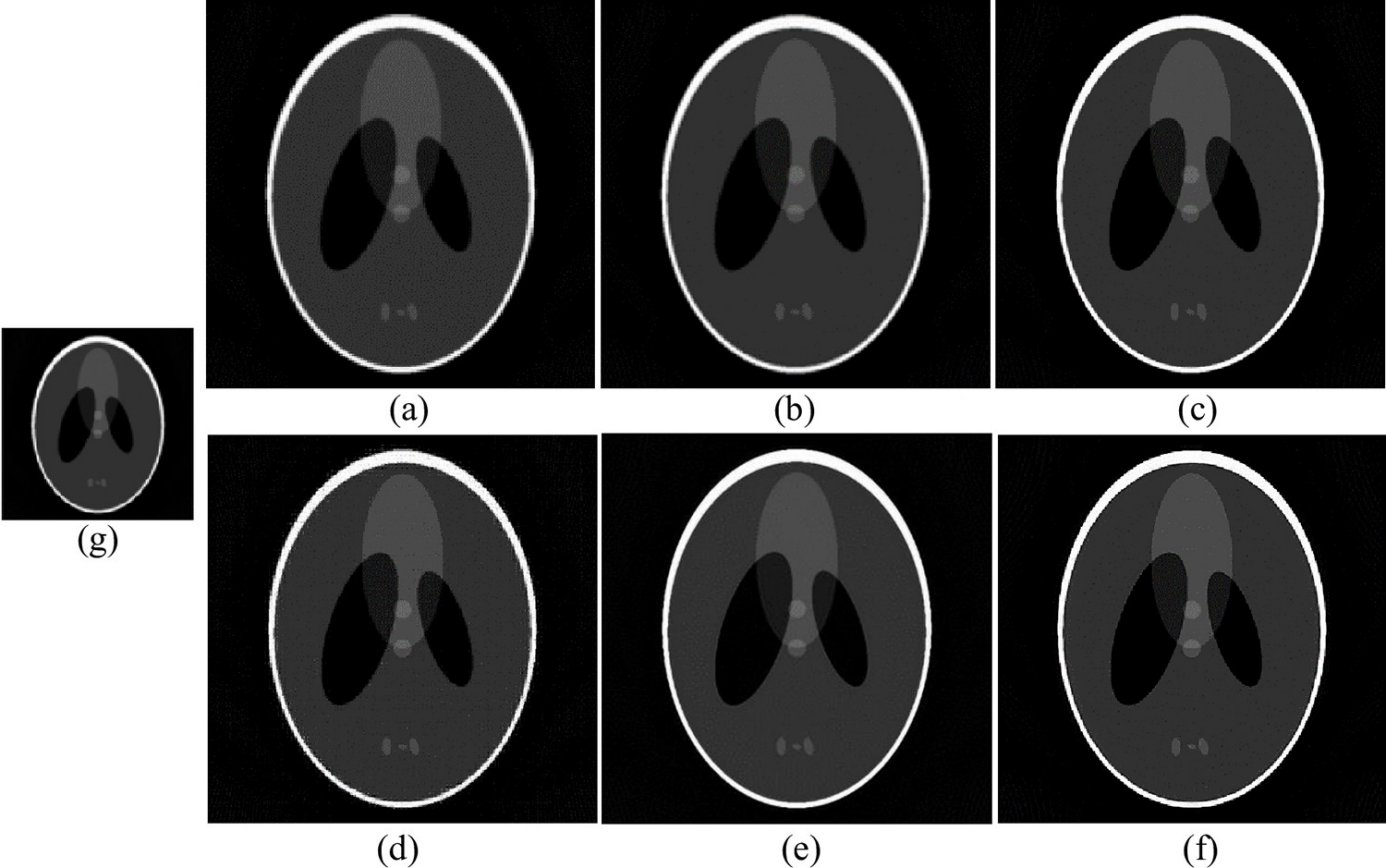
Improved sparse domain model algorithm compared with other image SR reconstruction algorithms. (g) Pictures to be reconstructed; (a) Nearest; (b) Spline; (c) Zeyde’s [33]; (d) Deeba [34];(e) SRCNN [13]; (f) Ours.

**Fig 7:**
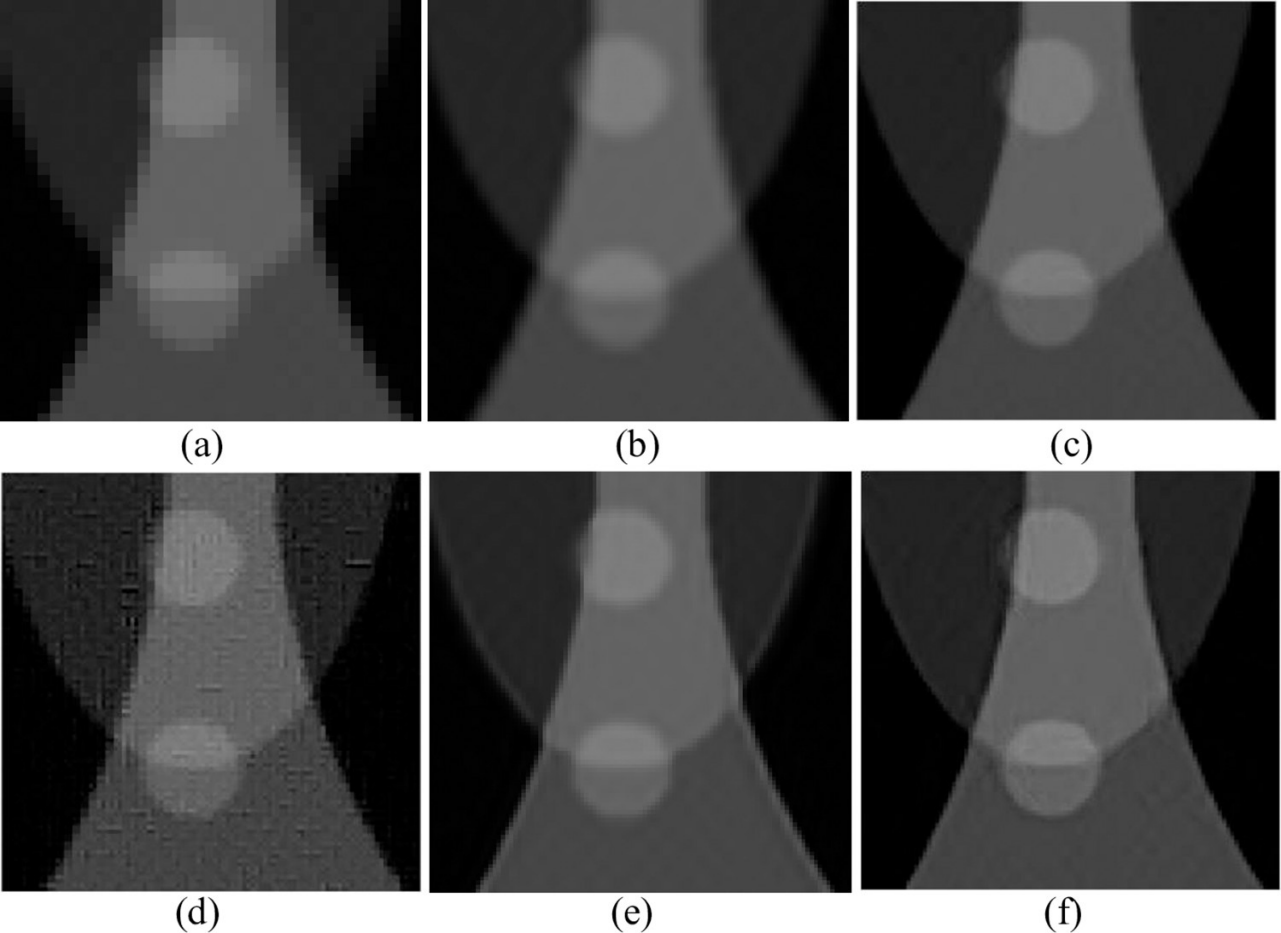
Pos. **(210:310,210:310).** (a) Nearest; (b) Spline; (c) Zeyde’s [33]; (d) Deeba [34]; (e) SRCNN [13]; (f) Ours.

**Fig 8:**
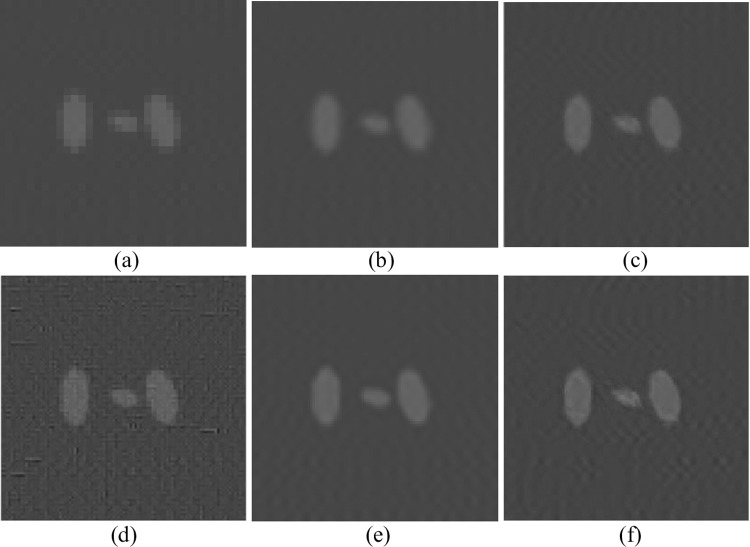
Pos. **(360:460,205:305).** (a) Nearest; (b) Spline; (c) Zeyde’s [33]; (d) Deeba [34]; (e) SRCNN [13]; (f) Ours.

In the experiment of changing parameters in the Shepp-Logan model, the dictionary size was 1000, the block size was set to 9, and the upscaling factor was set to 2. The training sample was a noisy SART reconstruction image, and the test sample was a low-precision FBP reconstruction image of 255*255. Firstly, blur and down sample the Shepp-Logan model to obtain Fig 6(G) as the image to be interpolated. Then perform SR reconstruction based on the interpolation method on an LR image, corresponding to nearest neighbor interpolation and spline interpolation in (a) and (b), respectively. Then, the original sparse domain model-based algorithm is used to obtain (c), the reference method [34] is used to obtain (d), the SRCNN-based algorithm is used to obtain (e), and the improved sparse domain model is used to obtain (f). The reconstruction results are shown in Fig 6, and some details are shown in Figs 7 and 8.

From Figs 7 and 8, it is evident that the texture structures generated by the nearest neighbor interpolation method, cubic interpolation method, and spline interpolation method are more blurry and cannot handle the detailed structures well. Compared with interpolation methods, reconstructed images based on the original sparse domain model and SRCNN reconstructed images significantly improve visual resolution. However, compared to other methods, the MeSR has clearer edges and more local structure processing, which is closer to the original image. To compare the effects of different reconstruction methods more clearly, in addition to subjective observations, Table 3 also lists the PSNR, entropy, and AG reconstructed by each algorithm.

**Table 3:** PSNR, entropy, and AG of different methods.

Method	Nearest	Spline	Zeyde’s [33]	Deeba [34]	SRCNN [13]	Ours
PSNR	23.8759	25.3466	29.3028	30.1150	30.2543	**31.0597**
Entropy	4.0731	4.1117	4.2038	4.2092	4.2804	**4.4939**
AG	2.2191	2.0732	2.2782	2.2071	2.2851	**2.7593**

The subjective vision and image quality indicators listed in Table 3 indicate that the proposed algorithm has achieved excellent performance, indicating that the method is feasible and effective in improving the SR reconstruction performance of medical images.

### Simulation model reconstruction

**Fig 9:**
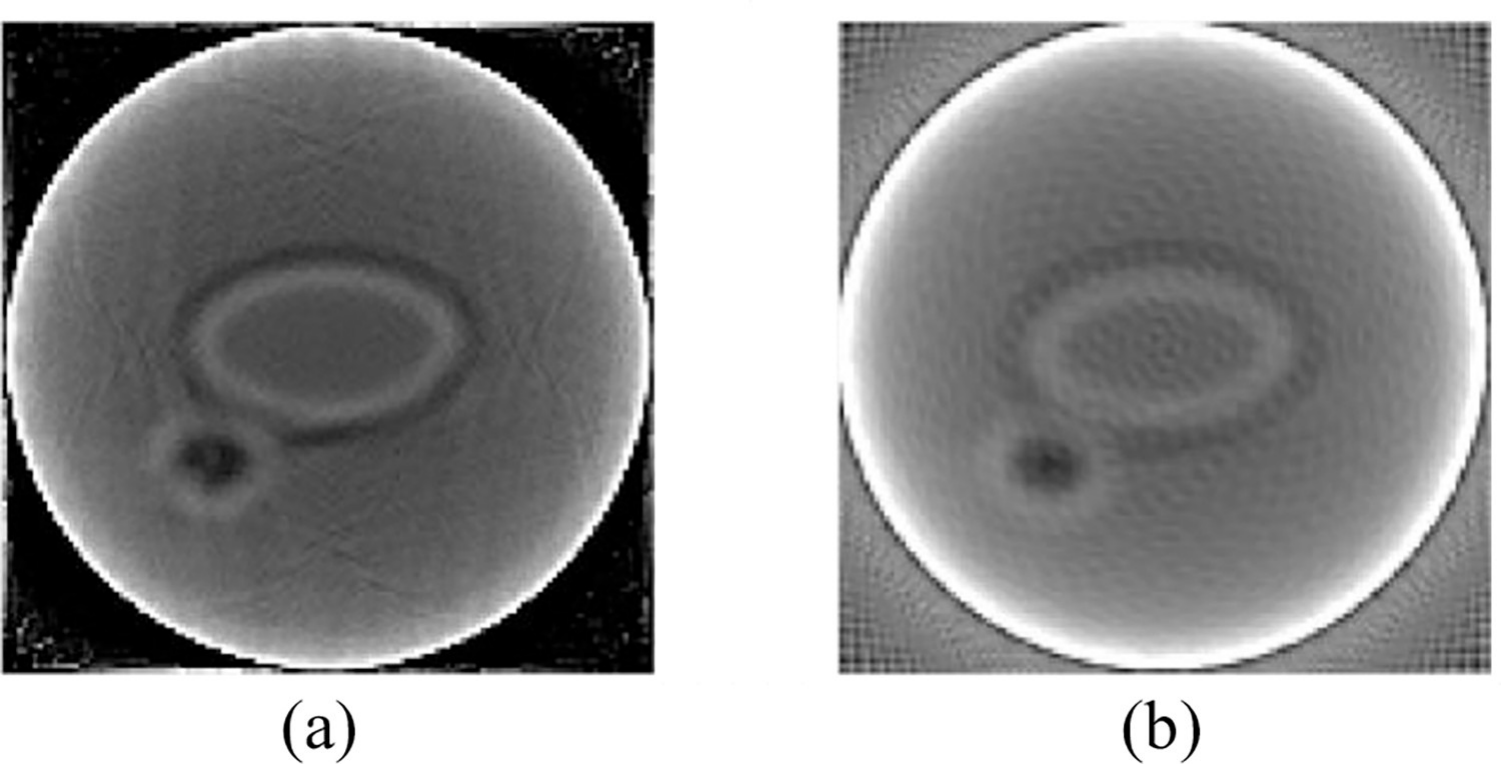
(a) SART image; (b) FBP image.

To better study the performance of the proposed MeSR reconstruction algorithm in the context of CMUT-based ultrasound imaging systems, this paper uses the k-wave toolbox [32] to simulate the propagation and reception of surface waves. The original digital breast model was used, including fibroadenoma, cancer, fat, and water. The corresponding attenuation values, sizes, and distributions are listed in Table 4. Use the rearrangement data method mentioned in 2.1 above for rearrangement, and then use the 128 angles SART imaging algorithm and FBP reconstruction method for reconstruction, respectively, to obtain Fig 9. In the experiment of a breast ultrasound tomography model based on CMUT, the dictionary size was set to 120, the block size was set to 9, and the magnification was set to 2.

**Table 4:** Parameters of the numerical breast phantom.

Tissue	*ρ* in *kg m*^−3^	*c* in *m s*^−1^	*α*_0_ in *dB*/MHz^*y*^ *cm*
Fat	950	1470	1.2
Fibroadenoma	1040	1515	0.7
Cancer	1070	1560	1
Water @ 26°C	1000	1500	1

The image presented using the SART algorithm has fewer artifacts and noise, and the image is clearer, while the image presented using the FBP algorithm has more artifacts. This simulation result confirms the above conclusion. We used SART images as training images for our algorithm and FBP images as test images for visual comparison with Nearest, Zeyde’s [33], Deeba [34], and SRCNN, as shown in Fig 9.

**Fig 10:**
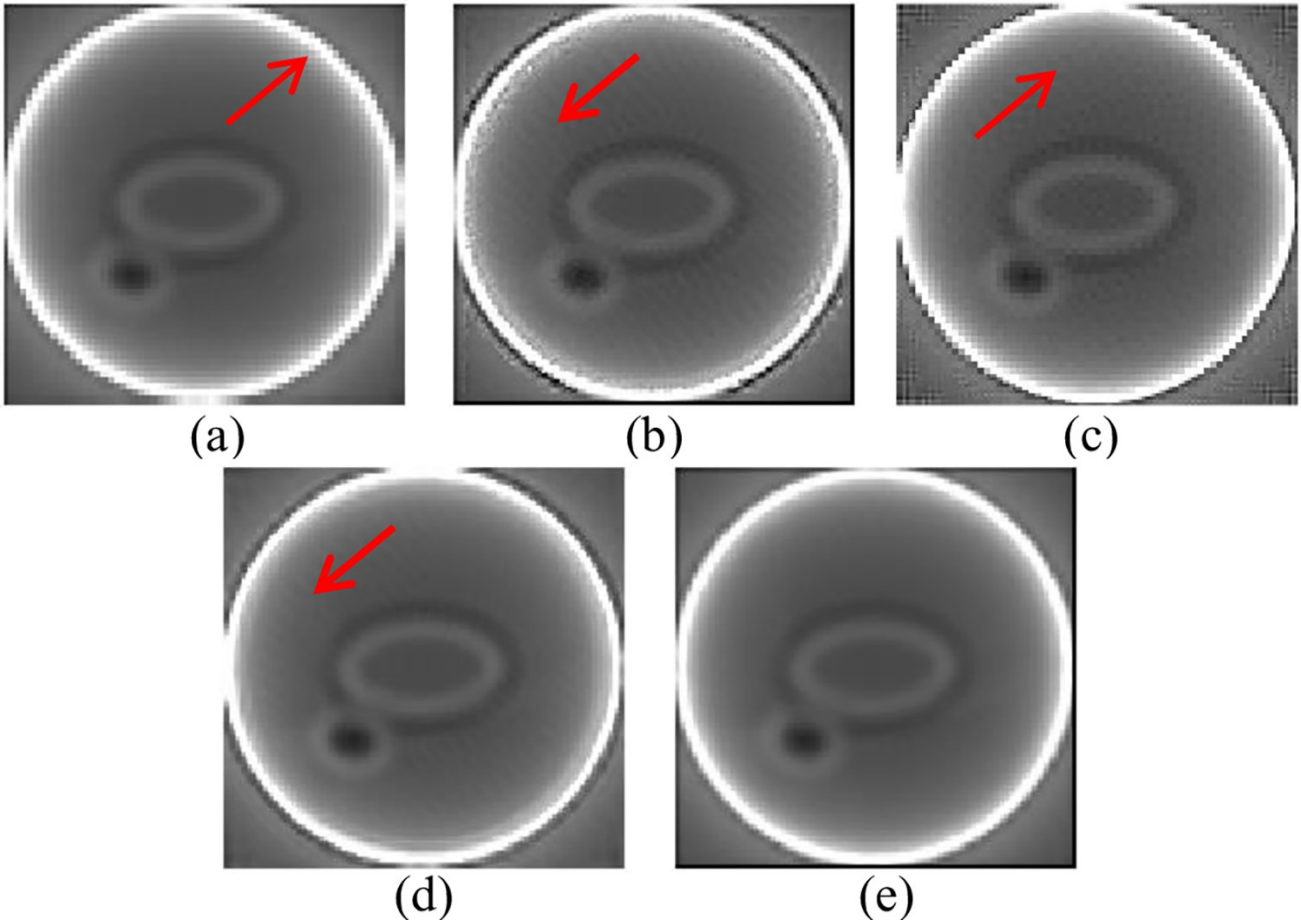
(a) Nearest; (b) Zeyde’s; (c) SRCNN; (d) Deeba [34]; (e) Ours.

We can see that the direction indicated by the red arrow in Fig 10(A) has a significant jagged effect, which cannot effectively preserve edge information. The direction indicated by the red arrows in Fig 10(B), 10(C) and 10(E) shows varying degrees of artifacts, while Fig 10(D) shows high image resolution and sound visual effects. Our Matlab code can be downloaded at the website: https://github.com/1997wzq/MeSR/tree/master.

## Conclusions

Due to issues such as machine noise or poor accuracy of reconstruction algorithms, medical image reconstruction involves raw noise, which may lead to traditional sparse domain methods learning about artifacts and noise in reconstructed images. o address this, researchers studied an improved sparse domain model called MeSR, which is a medical image SR reconstruction algorithm. This method can still use noisy images instead of clean ones as input images for the training phase, even if noisy reconstructed images are generated due to low precision reconstruction algorithms, machine equipment errors, or transmission errors, HR dictionaries can still be obtained. MeSR has been successfully applied to the Shepp Logan model and the breast ultrasound tomography system based on CMUT, achieving high performance. However, it has higher computational complexity compared to the original sparse domain model. In the future, further research will be conducted on parallel algorithms for single images and SR reconstruction of multiple projected images to achieve rapid reconstruction of 3D models.

## Supporting information

S1 Data (ZIP)
